# Boxing for Parkinson's Disease: Has Implementation Accelerated Beyond Current Evidence?

**DOI:** 10.3389/fneur.2019.01222

**Published:** 2019-12-04

**Authors:** Meg E. Morris, Terry D. Ellis, Dana Jazayeri, Hazel Heng, Andrea Thomson, Arun Prasad Balasundaram, Susan C. Slade

**Affiliations:** ^1^La Trobe Centre for Sport and Exercise Medicine Research, School of Allied Health, Human Services and Sport, La Trobe University, Bundoora, VIC, Australia; ^2^Healthscope North Eastern Centre, Ivanhoe, VIC, Australia; ^3^Center for Neurorehabilitation, Boston University College of Health and Rehabilitation Sciences, Sargent College, Boston, MA, United States

**Keywords:** Parkinson's disease, boxing, exercise, physical therapy, systematic review

## Abstract

**Background:** Exercise and physical activity are argued to promote neural plasticity in Parkinson's disease (PD), with potential to slow disease progression. Boxing for PD is rapidly growing in popularity.

**Objectives:** (i) To evaluate evidence on benefits and risks of boxing exercises for people living with PD and (ii) to appraise websites for evidence of global implementation of this intervention.

**Data Sources:** We searched AMED, CINAHL, Cochrane, EMBASE, EMCARE, Health and Medical Collection via ProQuest, MEDLINE, and PEDro electronic databases for the research literature. Websites were also searched for evidence of successful implementation of boxing for PD.

**Study Selection:** Published research and websites were considered if they reported data on adults with PD and boxing as an intervention.

**Data Extraction:** For the literature review, two reviewers independently extracted data on study characteristics and intervention content. Risk of bias was assessed with the PEDro scale and Joanna Briggs Checklist. We conducted a quality appraisal of websites using the QUality Evaluation Scoring Tool (QUEST).

**Data Synthesis:** Two studies, with a total of 37 participants, met the review eligibility criteria for the literature review. Risk of bias was low in these trials. Balance confidence, mobility, and quality of life were reported to improve with community-based boxing training programs delivered in 24–36 sessions over 12 weeks. PD medications were not always documented and some elements of the boxing interventions were incompletely reported against the CERT (Consensus on Exercise Reporting Template). Nine websites advocating boxing programs for PD were also evaluated. The QUEST analysis showed low-level quality, and little scientific evidence verifying findings, despite positive reports.

**Limitations:** In the published literature, findings were limited due to the small number of included studies and participants. Websites were numerous yet often lacked verifiable data.

**Conclusions:** Despite the recent growth in the popularity of boxing for PD and some positive findings, there is limited evidence of efficacy. Risks and disease-specific modifications have not been reported. Safety guidelines and health professional training are key considerations for implementation.

## Introduction

Although Parkinson's disease (PD) is progressive (1, 2) and currently has no cure (3), high intensity therapeutic exercises are argued to slow the rate of disease progression by promoting neural plasticity (4–7). Animal models of PD indicate that high dosage exercises might limit the depletion of dopamine producing neurons in the substantia nigra pars compacta and enhance adaptive mechanisms for dopamine and glutamate neurotransmission (8). They have also shown neuroprotective mechanisms induced by exercise to be linked to an increased production of brain-derived neurotrophic factor (BDNF), growth factors, and reduced free radicals (9). Specifically, high dosage running in rodent models of PD increases BDNF and neural generation in the hippocampus and reduces oxidative stress and dopaminergic loss (9, 10). Likewise, in humans with PD, Frazzitta et al. (4) reported that high intensity physical therapy incorporating gait training, strengthening and cueing for 2 hours a day for 4 consecutive weeks had neuroprotective effects on the dopaminergic system as well as increasing BDNF (4). Schenkman et al. (11) also showed high-intensity aerobic treadmill training in early PD to be feasible, with promise of neuroprotection.

One of the challenges faced by people with PD is how to sustain regular exercises over long time periods, given that they often live with the disease for 25–30 years. Interventions such as progressive resistance strength training (12–14), cueing (14–16), aqua therapy (17, 18), walking programs (19, 20), dancing (21–23), tai chi (24), and aerobic exercise (11), can assist people to move more easily and to enjoy a higher quality of life in the short term. However, people can find it difficult to sustain traditional exercises over the longer term and attrition can be a problem (25).

Boxing for PD has recently received increased attention across the globe (26, 27). As a form of high intensity exercise, it is argued that goal-based activities such as boxing can be engaging and accessible for people with chronic diseases (28). One of the challenges faced by physical therapists, health professionals and consumers is accessing the evidence for boxing for PD and knowing its suitability for the different phases of disease progression.

It is estimated that more than 3,000 people in North America, over 1,000 in Europe and more than 500 in the Asia-Pacific are currently participating in boxing for PD programs (29, 30). Boxing incorporates high-intensity exercise, with movements of all regions of the body in a weight bearing and aerobic context (31, 32). Either non-contact or as a contact sport, boxing movements can be performed in sitting, standing or as part of dynamic, complex movement sequences (31, 32). In able-bodied people, high intensity boxing programs performed for 50-minutes four times per week improved fitness, health and well-being (31). For boxing, as with any form of exercise therapy, there is a need to ensure safety, feasibility, and benefit. For people living with PD it is particularly important to understand the evidence and if there are risks, contra-indications, or adaptations needed, given that movement disorders, falls, and complex medication regimens are common (33).

Because boxing for PD has quickly grown in popularity, there is a need to understand whether the scientific evidence matches subjective reports of benefit, given the rapid global uptake. We investigated this by conducting (i) a review of literature reporting the evidence on boxing exercises for people living with PD (ii) a quality appraisal of websites with online health information on boxing for PD. We discuss the degree to which these concur.

## Materials and Methods

A review of the research literature was conducted informed by Cochrane guidelines, using the Cochrane handbook as a reference (34). The Preferred Reporting Items for Systematic Reviews and Meta-Analyses (PRISMA) guidelines enabled comprehensive reporting (35, 36). The protocol was registered on the International Prospective Register of Systematic Reviews (PROSPERO: CRD42018115122; http://www.crd.york.ac.uk/PROSPERO). All stages of analysis were conducted by two independent reviewers who met to reach consensus and consulted with a third reviewer when necessary. The review was conducted according to the published PROPSERO protocol with the addition of a search and appraisal of boxing for PD websites.

### Eligibility Criteria

The eligibility criteria form used to screen each research publication is given in Appendix 1 (Supplementary Material). Studies were included if participants were adults with a diagnosis of PD and the interventions included boxing, boxing exercises or boxing training. Interventions could be stand-alone or in combination with other forms of exercise or physical therapy. Studies could be of any design (randomized controlled trials, controlled clinical trials, quasi-experimental designs, pre-to-post-study designs, cohort studies, case-series, and case control studies). For inclusion, publications had to report an outcome such as disability, function, balance, falls (incidence, rate, or frequency), gait quality, gait speed, freezing, or quality of life. The trial reports also needed to include data measured at baseline, together with data obtained within or following the intervention period and to have quantitative raw data enabling statistical analysis. The study setting could include hospitals, outpatient settings, research laboratories, the home, residential care, or the community.

Published studies were excluded if they were conference abstracts, PhD theses, commentaries, editorials or expert opinions or if they investigated brain imaging, epidemiological, pharmacological or surgical interventions. Also excluded were studies where participants were diagnosed with conditions such as stroke, multiple sclerosis, traumatic brain injury, frailty, or non-movement neurological conditions.

### Data Sources and Searches

Eight electronic databases (AMED, CINAHL, Cochrane, EMBASE, EMCARE, Health and Medical Collection via ProQuest, MEDLINE, and PEDro) were searched using the following terms and synonyms: Parkinson's, Parkinson disease, PD, idiopathic primary parkinsonism, primary parkinsonism, shaking palsy, boxing, combat sports, punch, pugilism, amateur boxing from inception up until August 14, 2019. The databases were searched with comparable strategies using terms and search language adapted to the individual database format. The Medline search strategy is listed in Appendix 2 (Supplementary Material). Reference lists of the included studies were hand-searched and experts in the field of movement disorders were consulted.

### Study Selection

**Figure 1:**
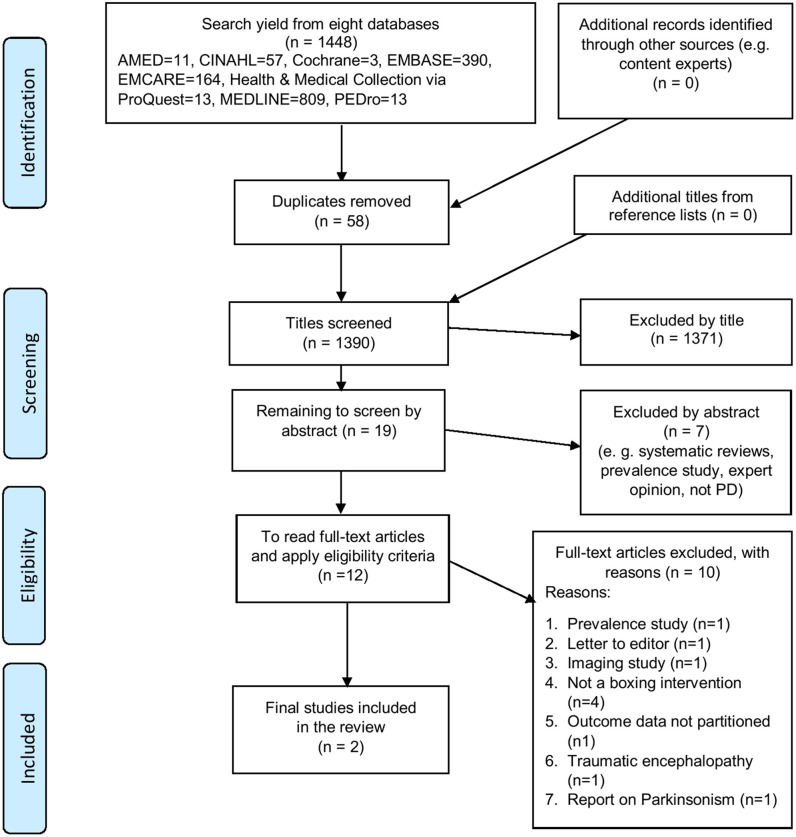
PRISMA-compliant flow diagram illustrating the process of study selection into the review (35, 36).

The search results were exported into a bibliographic management database (37). Initial screening of titles was performed by application of the *a priori* eligibility criteria (SCS, APB) and duplicates were deleted. Two independent reviewers (AT, APB) screened the titles and abstracts of remaining references and performed full-text review to identify studies that fulfilled the eligibility criteria. Disagreements were resolved through discussion, and a third reviewer (SCS) was consulted for confirmation and consensus. Study selection into the review is summarized in a PRISMA-compliant flow diagram (Figure 1).

### Method Quality/Risk of Bias Assessment

Studies included in the review were appraised for method quality by two independent reviewers (AT, APB) and SCS was consulted for confirmation and consensus. The Physiotherapy Evidence Database (PEDro) scale (38, 39) was applied to randomized controlled trials. A valid instrument was selected for other study designs, from the Joanna Briggs Institute Critical Appraisal Tools database (40). Each study was assigned a rating of high, moderate or low risk of bias according to the scoring matrix of each instrument.

### Data Extraction

Two reviewers (AT, APB) independently extracted data into a pre-tested spreadsheet under headings for study, participant and intervention characteristics and outcome data (Table 1). The data were independently screened and confirmed (SCS). Outcome data were extracted for short, medium and long-term follow up assessments. The boxing exercise intervention elements were independently extracted (AT, APB) into the Consensus on Exercise Reporting Template (CERT) (43, 44) and confirmed by SCS.

**Table 1:** Characteristics of trials included.

**First Author (year), country, setting**	**Study design**	**Sample size (M, F), mean age (SD)**	**Disease duration months (range or SD)**	**Interventions summary (duration, dosage, and frequency)**	**Medications**	**Co-morbidities**	**Dependent variables, measurement tools**
Combs et al. (41), USA, Community Intervention group: community classes led by personal trainers Control group: university campus; 2 students supervised by physical therapist	RCT	31 (M: 21, F: 10) Boxing: *n* = 17 Exercises: *n* = 14 *Boxing group* 68.0 years (31.0) *Exercise group* 66.5 years (28.0)	*Boxing* Median 50 (range 99.0) *Exercise group* Median 41.5 (range 182)	*Boxing group:* 1. 15-min warm up exercises (seated) 2. 45–60 min boxing circuit training, including functional, endurance, and punching exercises *Exercise group:* 1. 15-min warm up exercises (seated) 2. strengthening, endurance ans balance exercises 3. 15-min cool down exercises Dosage: 24–36 sessions of 90 min over 12 weeks	Not reported	Not reported	1. Balance 2. Balance activities confidence 3. Mobility 4. Gait speed 5. Walking endurance 6. Quality of life Measurement tools: 1. BBS 2. ABC 3. TUG and dTUG 4. Timed gait 5. 6MWT 6. PDQL
Combs et al. (42) USA, Community Delivered by professional boxers who were also personal trainers	Case series	6 (M: 6, F: 0) 60.17 years (10.26)	Mean 28.67 (*SD* 24.34)	24–36 boxing sessions over 12 weeks; optional continuation. 1. 20-min warm up: breathing and stretching exercises 2. 45–60 min circuit training including functional and endurance and punching exercises, pushups, skipping, treadmill, cycling, running (3 min bouts with 1 min rests) 3. 15–20 min cool down focusing on breathing, stretching and strength	Dopamine replacement therapy in 4 participants	Not reported	1. Balance 2. Balance activities confidence 3. Mobility 4. Gait speed, cadence, stride length, step width 5. Walking endurance 6. Quality of life 7. Disability Measurement tools: 1. BBS and FRT 2. ABC 3. TUG 4. Timed gait 5. 6MWT 6. PDQL 7. UPDRS

*BBS, berg balance scale; ABC, activities-specific balance confidence scale; TUG, timed up and go; dTUG, dual-task TUG; 6MWT, 6 min walk test; PDQL, Parkinson's disease quality of life scale; FRT, functional reach test. M, male; F, female; PT, physical therapist; RCT, randomized controlled trial*.

### Data Analysis

Summary statistics were used for data analyses where possible, and included descriptive, textual, nominal, categorical (including dichotomous and other), ordinal, ratio, or metric (continuous) types. For the categorical data, the relative risks and the odds ratios, with 95% confidence intervals (CIs), were calculated where possible (**Table 4**). For continuous data, mean difference scores, with 95% CIs, were calculated if the included studies used the same outcome measures. Continuous outcome data were analyzed using the standardized mean difference if studies reported the same outcome but with an alternative measurement instrument (e.g., function, falls, quality of life). Meta-analysis occurred where data from randomized controlled trials could be pooled and were described in a narrative format when pooling was not possible.

### Strength of Evidence

The Grading of Recommendations Assessment, Development and Evaluation (GRADE) guidelines were used to determine the overall quality of evidence for the outcomes of interest (45). The overall quality of evidence can be influenced by study design, method quality, and imprecise data (46).

### Website Search and Evaluation

A supplementary search of websites was conducted with methods informed by Briscoe (47) and Stansfield et al. (48). There are no systematic review or Cochrane guidelines for website searches and evaluations. The search was completed by three independent reviewers on August 6, 2019. The search engines used were “Google,” “Google Scholar,” “Bing,” and “Duckduckgo” and the search terms, and synonyms, included Parkinson's disease, Parkinson disease, Parkinsonism, Parkinson, PD, Shaking Palsy, Movement Disorders, Boxing, Box, Boxercise, Ready Steady Boxing, Boxing for Parkinson's, PD Warrior, Physiotherapy, and Physical Therapy. The World Parkinson Coalition and the International Parkinson and Movement Disorder Society were contacted for their lists of PD organization websites. Eligible websites were those that included data on adults with PD and boxing exercises as an intervention. Websites were excluded if they could not be translated into English; were online versions of newspaper articles, editorials, blogs, or advertisements; business promotions; web links to sub-sites; or websites with limited information, such as sites indicating only event details. Two independent reviewers conducted the initial screening and a final number of included websites was reached at a consensus meeting. Final website appraisal was conducted on August 14, 2019.

The QUality Evaluation Scoring Tool (QUEST) tool was chosen for the website appraisal because it has been confirmed to be a valid, reliable appraisal tool for websites (49). It has a scoring matrix and range of possible total scores of 0–28 with a higher score indicating better quality. Two independent reviewers examined the websites for each QUEST component and provided scores for each section. The reviewers then met to reach consensus with a third reviewer acting as an arbiter.

## Results

The literature review results are presented textually, with flow charts, summary tables, statistical analysis (and meta-analysis where possible), and narrative summaries. Meta-analysis and subgroup analysis were not performed due to a lack of data. From a database search yield of 1,448 titles, two studies met the eligibility criteria and were included in the review (41, 42). Figure 1 shows a PRISMA-compliant flow chart demonstrating selection of studies into the review. The final excluded studies, with reasons for their exclusion, are listed in Appendix 3 (Supplementary Material).

### Study Characteristics

A total of 37 participants with PD were investigated in the two included studies, which were both led by Combs et al. (41, 42). The randomized controlled trial by Combs et al. (41) included 21 males and 10 females with a mean age of 67.2 years and a mean disease duration of 45.7 months. The case-series study included six males with a mean age of 60.2 years and a mean disease duration of 28.7 months (42). Both reported key outcomes such as balance, gait speed, walking endurance, mobility and quality of life and used a range of measurement instruments (Table 1).

### Method Quality Assessment Results

The method quality assessment of the randomized controlled trial (41) indicated a low risk of bias with a PEDro score of 7/10 (Table 2). Although there was a low risk of bias for the case series (42) there was the possibility of selection bias because recruitment was not consecutive (Table 2).

**Table 2:** Method quality assessment of included studies.

**Randomized controlled trial (PEDro scale)** **(****38**, **39****)**											
First author, year	Random allocation	Concealed allocation	Baseline—similar	Blinded participant	Blinded therapist	Blinded assessor	Adequate follow-up	ITT	Between- group analysis	Outcome measure data—point estimates and variability	**Score/10**
Combs et al. (41)	Y	Y	Y	N	N	Y	N	Y	Y	Y	7/10
**Case series (JBI appraisal tool)** (https://joannabriggs.org/critical_appraisal_tools)											
First author, year	Eligibility criteria	Standard measures	Diagnostic criteria	Consecutive inclusion	Complete inclusion	Demographic data	Clinical data	Results reported	Setting	Statistical analysis	**Score /10**
Combs et al. (42)	Y	Y	Y	N	N	Y	Y	Y	Y	Y	8/10

### Exercise Intervention Elements

Some elements of the boxing interventions were incompletely reported in both studies. For the randomized controlled trial (41) 6/19 CERT items were described in detail which was adequate for replication, while for the case series (42) 8/19 CERT items were described in replicable detail (Table 3). Neither of the studies comprehensively reported the following boxing exercise elements according to the CERT analysis: exercise equipment, adherence and motivation strategies, decision rules for intervention starting levels and progression, whether there was a home program and non-exercise components (e.g., stretching, education), or individual tailoring of exercise elements. Both studies comprehensively described instructor qualifications, exercise supervision, whether the boxing interventions were conducted individually or in a group, the exercise setting progression, and the intervention dosage and duration.

**Table 3:** Results for each CERT item.

**CERT item**	**Combs et al. (41)**	**Combs et al. (42)**
1. Equipment	X	X
2. Instructor qualifications	✓	✓
3. Individual/Group	✓	✓
4. Supervision	✓	✓
5. Adherence	X	X
6. Motivation	X	X
7a. Progression rule	X	X
7b. Progression described	✓	✓
8. Exercise detail replicable	X	✓
9. Home program	X	X
10. Non-exercise components	X	X
11. Adverse events	X	✓
12. Setting	✓	✓
13. Intervention described	✓	✓
14a. Generic/tailored	X	X
14b.Tailoring method	X	X
15. Starting level	X	X
16a. Fidelity measured	X	X
16b. Fidelity described	X	X

*✓, reported CERT item. X, did not report CERT item*.

### Data Analysis

For the randomized controlled trial (41) effect sizes were reported at the end of the intervention and were without 95% confidence intervals or raw data. There were no long-term follow-up data to report retention of training. Effects were negligible in size or comparatively small for balance, functional mobility, gait speed, and quality of life. Effects were moderately strong for gait endurance and comparatively large for activity specific balance confidence [(41); Table 4].

**Table 4:** Data analysis for randomized controlled trial (41).

**First author, year**	**Outcome measure**	**Between group differences (favoring boxing)**	**Time-points**
Combs et al. (41) Randomized control trial *n* = 31 (Intervention = 17, Control = 14) Boxing exercises vs. traditional exercises 12-week intervention	BBS higher better ABC (%) higher better TUG (sec) higher better dTUG (sec) higher better Gait Speed (m/s) higher better 6MWT (m) higher better PDQL higher better	Effect Size (ES); (Means, SD and Confidence Intervals not reported) BBS: 0.28 (small ES) ABC: 0.97 (large ES) TUG: 0.09 (small ES) dTUG: 0.07 (small ES) Gait Speed: 0.28 (small ES) 6MWT: 0.65 (moderate ES) PDQL: 0.15 (small ES)	1.Baseline 2. 1-week post-intervention 3. No medium or long-term follow-up

*BBS, berg balance scale; ABC, activities-specific balance confidence scale; TUG, timed up and go; dTUG, dual-task TUG; 6MWT, 6 min walk test; PDQL, Parkinson's disease quality of life scale*.

The case-series study by Combs et al. (42) had three people with mild PD and three with moderate to severe PD. For those with mild PD, boxing exercises had little effect on balance and varying degrees of change for activities-specific balance confidence, although quality of life increased a small amount for patient 1 and patient 2 (42). Stride length increased from baseline to the 36-week post-test. There were also improvements in walking endurance on the 6MWT. For the three people with more severe PD, there were some small improvements in balance from baseline to the 36-week post-test. Activities-specific balance confidence, TUG mobility and gait speed also increased. There were no adverse events reported for this case series (42).

### GRADE Evidence Summary

The overall summary of evidence was not generated because of the small number of studies of heterogenous design (*n* = 2).

### QUEST Website Evidence Appraisal

The initial yield from the online search of boxing for PD and PD organizations was 448 websites. Of these, 418 were excluded because they were either business advertisements or promotions, personal testimonials, event lists, blogs, newsletters, unreferenced summaries, media releases or presented in non-English languages. After duplicates were deleted, there were 29 websites, which were evaluated for eligibility at a consensus meeting. On closer examination, 20 were excluded because they were news media, blogs, event lists or referred to general exercises and not boxing. Nine websites were included in the final analysis (26, 27, 29, 30, 50–54).

Two independent reviewers applied the QUEST tool to the final nine included websites to appraise global boxing implementation for PD (49). Consensus was reached with a third reviewer (SS) acting as an arbiter. This appraisal indicated that websites were variable, and generally poor in quality in relation to comprehensive, validated boxing interventions, scoring 3/28 to 20/28 (Table 5). The websites advocated boxing as beneficial for people with PD and presented boxing exercise classes in locations across the USA, Europe, Australia, and Asia. Many provided testimonials and they frequently endorsed boxing for PD with limited reference to evidence-based research. Other important information such as the date of publication or qualifications of the authors of the website were usually not provided (Table 5). This analysis indicated that higher quality online health information is required to support the efficacy of boxing for people living with PD.

**Table 5:** QUEST appraisal of included websites.

	**Website URL**	**Organization country of origin**	**Authorship e.g., author name and qualifications**	**Attribution e.g., expert source, research findings**	**Type of study (for articles scoring >6 for attribution)**	**Conflict of interest e.g., endorsements or promotions**	**Currency (publication date)**	**Complementarity (degree of support for consumer-medical relationship)**	**Tone (full or cautious support)**	**Total 0–28 (higher is better)**
1.	https://www.rocksteadyboxing.org	USA and international	0	9	2	0	2	1	3	17
2.	https://kopd.com.au/	Australia	0	3	N/A	0	0	1	3	10
3.	http://www.knockoutparkinsons.com	USA	0	6	1	0	0	1	0	7
4.	https://punchingoutparkinsons.org/	USA	1	0	N/A	0	2	0	0	3
5.	https://www.michaeljfox.org	USA	2	6	2	0	2	1	3	16
6.	https://www.parkinson.org/	USA	0	0	N/A	0	2	1	3	6
7.	https://www.epda.eu.com/	Europe	1	6	1	0	2	1	3	14
8.	https://www.wiparkinson.org/	USA	1	9	1	0	2	1	6	20
9.	https://shakeitup.org.au	Australia	0	6	1	0	2	1	3	13

## Discussion

Despite the promising affirmations on worldwide websites about boxing for PD [e.g., (30, 50–52)], the research literature is currently confined to one small randomized trial (41) and one small case series evaluation (42). Both of these publications were from the same research group. There is a marked mismatch between the strength of the positive rhetoric on some online information websites about boxing for PD and the actual research evidence of benefits and limitations (33). The lack of evidence does not mean that boxing is not helpful for people with Parkinsonism. Rather, the benefits, precautions, contraindications, and limitations have not yet been verified.

In relation to PD and related conditions, what is not known is which forms of exercise, physical activity, and movement rehabilitation are most beneficial for each individual across the different stages of disease progression (55). PD progresses at different rates in individuals over periods of 5–30 years (56). It is possible that high intensity aerobic exercises incorporating boxing could be most beneficial in the early to middle stages of disease progression, and less appropriate after very many years or at end-stage disease when sometimes the focus can be on palliative care (57). Also not clear is what dosage of high intensity aerobic exercise (such as boxing) is required in the prodromal phase before diagnosis, or in the early and intermediate stages of PD (58). Moreover, there is little clarity on how many consecutive weeks, times per week or minutes per boxing session are needed to gain benefits, or how boxing exercises should be modified to be safe and therapeutic. Comprehensive descriptions of the components of the boxing interventions need clarification. For example, description of the type of punches (jab, cross, hook, and upper cut), the number of unilateral or bilateral punches in each sequence and whether the participants sit or stand, use a punching bag, shadow box or spar with a partner would enable replication. Contraindications and precautions need documentation, as well as guidelines for trainers and therapists about how to modify boxing exercises according to co-morbidities, fitness, and the locus of the PD medication cycle (16). In addition, sometimes classes incorporating boxing for people with PD are not actually high intensity and could be seen as beneficial in other ways, such as for balance, anticipatory control, trunk rotation, or for social engagement.

Arguably, there needs to be a continuum of care provided by healthcare professionals to prescribe optimal boxing exercise programs for the different stages of disease progression, based on the most current evidence. Partnerships with fitness professionals in the community to implement evidence-based exercise programs over the long-term could also help to build crossroads between specialists and community-based care (59). This could, for example, incorporate periodic re-assessments and program revisions from physical therapists to ensure that community boxing exercise programs are tailored to individual needs and adapt to changes in movement disorders and non-motor symptoms.

Boxing brings an advantage of being community-based, accessible and a social form of vigorous and sustained exercise. Traditionally, therapeutic exercises for people with PD were delivered in the context of hospital or home-based movement rehabilitation programs (13, 17, 25). Often limited to 2–6 weeks (60, 61), traditional therapies of this nature had comparatively high levels of attrition. More recently, attention has shifted to physical activities that are high dosage, sustained over the long-term and are engaging, enjoyable and motivating for people living with PD (17, 62). Boxing arguably fulfills these criteria. Nevertheless, when reviewing the ingredients of the boxing programs in Table 1, it is apparent that a considerable amount of time was often spent on other forms of exercise, such as stretching, push-ups, strength training, balance and treadmill training. In some cases, boxing classes appeared to be more analogous to a comprehensive gymnasium exercise program, incorporating a range of different forms of therapeutic physical activities, with the added benefits of social interaction. The relative benefits of the boxing elements compared to other program ingredients (e.g., strengthening, stretching, balance, mobility, aerobic training, agility training, endurance training, and social connectedness) needs to be determined so that improvements can be accurately attributed, and consumers can make informed decisions about participation. Agility training (6, 63) has recently been shown to reduce the rate of disease progression in PD and the extent to which boxing incorporates these elements to optimize outcomes awaits verification.

There were some limitations of the review and website analysis. We only evaluated material presented in English and reported findings after 1990. The two included research studies were from the same research team. The gray literature was not searched for the review and personal opinions and testimonials were excluded from website inclusion. We did not perform an economic evaluation of boxing for PD. A strength is that this evaluation provides the first worldwide report of evidence and uptake of boxing for people living with this progressive and comparatively common neurological disorder.

To conclude, boxing for individuals with PD is popular, with more than 4,500 participants worldwide (27, 29, 30). Our review indicates that implementation has accelerated beyond the current research evidence. Given the rapid uptake, there is an acute need for randomized trials to test effectiveness, efficacy, feasibility and safety, and to ensure evidence-based application. Health professional and boxing instructor training are also key to ensuring necessary modifications to delivery, given that movement disorders, balance problems and co-morbidities can be experienced by people living with PD. Contraindications and precautions related to boxing for PD need to be derived, validated and implemented globally.

## Data Availability Statement

All datasets generated for this study are included in the article/Supplementary Material.

## Author Contributions

MM conceived the idea for the study. MM, SS, DJ, HH, AT, and AB were responsible for the study design, study implementation, writing, and manuscript revisions. SS was responsible for administering the literature search and for the CERT analysis. All authors (MM, SS, TE, DJ, HH, AT, and AB) have contributed to read and approved the final manuscript.

### Conflict of Interest

The authors declare that the research was conducted in the absence of any commercial or financial relationships that could be construed as a potential conflict of interest.
