# Supplementary material for: Boxing for Parkinson's Disease: Has Implementation Accelerated Beyond Current Evidence?
Source: Front Neurol. 2019 Dec 4;10:1222. doi: 10.3389/fneur.2019.01222 (PMC6904341; doi:10.3389/fneur.2019.01222)
Supplement: Supplementary file 1 [file Table_1.DOCX]

**Appendix 1: Eligibility criteria**

**Title (first author, year):**

| **Inclusion criteria** | | **Yes** | **No** |
| --- | --- | --- | --- |
| - Studies published in peer-reviewed journals - English language - Available in full-text - Multiple intervention studies could be included where the effects of co-interventions could be partitioned from boxing | |  |  |
| *Study Design*   - Any study design e. g. randomised controlled trials, quasi-experimental, pre-post, cohort, case-series and case-control studies | |  |  |
| *Population*   - Adults with a diagnosis of Parkinson’s disease | |  |  |
| *Intervention*   - Boxing, boxing exercises or boxing training as either as a standalone intervention or in combination with other forms of exercise or physiotherapy | |  |  |
| *Setting*   - Hospital, home, residential care, community-based or research laboratory | |  |  |
| *Data*   - Outcome measure data reported for baseline, post-intervention and follow-up measurement - Quantitative data such as point estimates | |  |  |
| **Exclusion criteria** | |  |  |
| - Conference papers/abstracts, PhD theses, commentaries, editorials, expert opinion - Study participants diagnosed with stroke, multiple sclerosis, traumatic brain injury, motor neuron disease and cerebral palsy - Studies with brain imaging alone, epidemiology of Parkinson’s disease, pharmacology or surgical procedures, or other types of exercise interventions | |  |  |
| **INCLUDE** | **EXCLUDE** | | |
| - For inclusion, articles must be rated as ‘YES’ for all inclusion criteria and have no exclusion criteria - Proceed to data extraction and method quality assessment | |  |  |

**REASON FOR EXCLUSION:**

**Appendix 2: Search strategy**

1. *Data sources:* The following databases were searched for eligible studies: AMED, CINAHL, Cochrane, EMBASE, EMCARE, Health and Medical Collection via ProQuest, MEDLINE, and PEDro.
2. *Search Strategy:* The following search terms, which included key words and medical subject headings (MeSH) were used.

*Note:* keywords are italicised; medical subject headings (MeSH) are emboldened; and Boolean operators are capitalised)

1. (*parkinsons* OR *'parkinson disease'* OR *'idiopathic primary parkinsonism'* OR *'primary parkinsonism'* OR *'hypokinetic rigid syndrome'* OR *'paralysis agitans'* OR *'shaking palsy'* OR *PD*).mp. [mp=title, abstract, original title, name of substance word, subject heading word, floating sub-heading word, keyword heading word, protocol supplementary concept word, rare disease supplementary concept word, unique identifier, synonyms]

2. (*boxing* OR *'combat sports'* OR *punch** OR *pugilism* OR *strik** OR *'amateur boxing'*).mp. [mp=title, abstract, original title, name of substance word, subject heading word, floating sub-heading word, keyword heading word, protocol supplementary concept word, rare disease supplementary concept word, unique identifier, synonyms]

3. exp PARKINSON DISEASE/

4. exp BOXING/

5. 1 OR 3

6. 2 OR 4

7. 5 AND 6.

**Appendix 3: Final excluded papers and reasons**

| **First Author Year** | **Title** | **Reasons for exclusion** |
| --- | --- | --- |
| Betti, 1969 | Pugilistic encephalopathy. Acta Neurologica Latino Americana. 15(1), 47-51. | Traumatic encephalopathy, not PD |
| Bhidayasiri, 2012 | Boxing and Parkinson disease: a link or a myth? An 18F-FDOPA PET/CT study in retired Thai traditional boxers. Parkinsonism & Related Disorders. 18(5): 694-696. | Prevalence study and not testing an intervention |
| Chaudhuri, 1995 | Magnetic resonance spectroscopic study of parkinsonism related to boxing. J Neurology, Neurosurgery & Psychiatry. 59(5): 561-562. | Letter to editor |
| Davie, 1995) | Magnetic resonance spectroscopic study of parkinsonism related to boxing. J Neurology, Neurosurg Psych. 58(6): 688-691. | Imaging study and not a boxing intervention |
| Fertl, 1993 | Physical activity and sports in patients suffering from Parkinson's disease in comparison with healthy seniors. J Neural Transmission - Parkinson’s Disease Dementia Section, 5(2):157-161. | Physical activity and not a boxing intervention |
| Frazzitta, 2009 | Rehabilitation treatment of gait in patients with Parkinson’s disease with freezing: a comparison between two physical therapy protocols using visual and auditory cues with or without treadmill training. Move Disorders. 24(8): 1139-1143. | Intervention was treadmill training and not a boxing intervention |
| Friedman, 1989 | Progressive parkinsonism in boxers. Southern Medical J. 82(5), 543-546. | Case report on Parkinsonism |
| Goncalves, 2014 | Effects of using the Nintendo Wii Fit Plus platform in the sensorimotor training of gait disorders in Parkinson’s disease. Neurol Internat. 6: 5048. | Outcome data cannot be partitioned for “rhythmic boxing” |
| King, 2009 | Delaying mobility disability in people with parkinson disease using a sensorimotor agility exercise program. Phys Ther. 89(40): 384-393. | Intervention was agility exercise and not boxing |
| van Vaerenbergh, 2003 | The influence of rotational exercises on freezing in Parkinson's disease. Functional Neurol. 18(1): 11-16. | Not a boxing intervention |
